# Supplementary material for: Exploring epidemic control policies using nonlinear programming and mathematical models
Source: PLoS Comput Biol. 2026 May 6;22(5):e1014238. doi: 10.1371/journal.pcbi.1014238 (PMC13170968; doi:10.1371/journal.pcbi.1014238)
Supplement: Attachment — Submitted filename: MB_report.pdf [file pcbi.1014238.s004.pdf]

Title: Exploring epidemic control policies using nonlinear programming and mathematical models

1. Summary of the Research

This manuscript investigates the application of direct optimal control methods, particularly nonlinear programming (NLP) using JuMP and IPOPT, to a series of epidemiological ODE models. The authors compare indirect methods (Pontryagin's Maximum Principle, forward-backward sweep) with direct discretization and solve several case studies, including lockdown optimization, "flattening the curve," vaccination strategies, and a dengue transmission model. The paper aims to demonstrate that direct NLP methods are practical, accessible, and flexible for epidemic policy optimization, especially relative to traditional indirect approaches.

The topic is potentially valuable, particularly given the increased interest in optimization for epidemic response during the COVID-19 pandemic. The manuscript is well written, and the examples are clear and reproducible.

However, the current version reads more like tutorial or software demonstration rather than a research article. The examples rely on low-dimensional "toy" systems, and important issues such as discretization robustness, scalability, and real-world policy constraints need further discussion. The use of extremely simplified models, the absence of rigorous numerical analysis, and the lack of engagement with the real complexities of epidemic control significantly limit the impact. In its present form, the novelty is modest and several essential issues remain insufficiently addressed.

Overall recommendation: Major revisions to clarify the manuscript's contribution and acknowledge limitations.

2. Examples and Evidence

Major Issues (must be addressed)

1. **Clarify the novelty and contribution.**
   JuMP, IPOPT, and direct NLP methods are not new to optimization or epidemiology. The paper's contribution lies in accessibility, reproducibility, and translation to practice. This should be stated explicitly in both the Introduction and Discussion to avoid overstating novelty.

2. **Discuss scalability beyond toy models.**
   All demonstrated models are low-dimensional (SIR variants, a 7-compartment dengue model), deterministic, and idealized. These settings do not represent the

challenges that make optimal control in epidemiology difficult (e.g., parameter uncertainty, non-convex feasible sets, delays, spatial structure, stochasticity).

The authors must explain why these simplified models are sufficient to support their claims, or else add at least one more realistic high-dimensional example.

3. **Address realism of interventions.**
   Some of the computed optimal controls (e.g., continuously varying lockdown intensity) are unrealistic in practice. The manuscript should acknowledge common policy constraints such as:
   - minimum/maximum intervention durations,
   - delays between decision and implementation,
   - capacity limits for vaccination.
     A brief discussion would help contextualize results for policy audiences.

4. **Lack of numerical analysis or solver robustness evaluation.**
   IPOPT's performance is highly sensitive to scaling, sparsity patterns, stiffness, constraint curvature, and discretization choices.
   The manuscript provides no analysis of:
   - sensitivity to timestep dt,
   - solver tolerance settings,
   - convergence failures,
   - constraint feasibility.
     Without this, the claim that the method is "robust" is unsubstantiated.

5. **Discretization choice (Euler) is inadequate without justification.**
   Euler's method performs poorly under moderate stiffness or when dt is not extremely small.
   The authors must justify its use or compare with more appropriate schemes (e.g., RK4, backward Euler, collocation). At minimum, they should report timestep sensitivity.

6. **No comparison with modern control approaches.**
   The Discussion briefly mentions differentiable ABMs but does not meaningfully engage with:
   - Model Predictive Control (MPC),
   - Reinforcement Learning,
   - Hybrid direct-indirect methods,
   - Deep learning-based optimization (e.g., DQN, policy gradients).
     Given current trends, this omission is noticeable.

Minor Issues (would improve clarity but do not affect conclusions)

1. Clarify whether parameters in SIR cases were chosen arbitrarily or based on literature.
2. Add brief comments on solver settings (tolerances, scaling), since IPOPT performance can depend on these.
3. Expand related work beyond a few COVID-era studies; optimal control in epidemiology has a longer history.
4. Provide a short statement on how non-convergence was handled (e.g., feasibility issues, solver failure modes).

Mohamed Bakheet, PhD
