# Supplementary material for: Exploring epidemic control policies using nonlinear programming and mathematical models
Source: PLoS Comput Biol. 2026 May 6;22(5):e1014238. doi: 10.1371/journal.pcbi.1014238 (PMC13170968; doi:10.1371/journal.pcbi.1014238)
Supplement: Attachment — Submitted filename: Resp_to_review_MontesOlivas_etal_2026.pdf [file pcbi.1014238.s007.pdf]

February 17, 2026

Dr Sandra Montes-Olivas
Researsh Fellow, Mathematical Modelling of Infectious Diseases
Dept of Infectious Diseases Epidemiology & Dynamics
London School of Hygiene & Tropical Medicine
Email: sandra.montes-olivas@lshtm.ac.uk
Keppel Street, London, WC1E 7HT

Professor Joseph T. Wu
Academic Editor
PLOS Computational Biology

Dear Professor Wu,

Thank you for the opportunity to revise our paper titled ‘Exploring epidemic control policies using nonlinear programming and mathematical models’. We are grateful to both reviewers for their thorough and constructive feedback, which has significantly improved the quality and clarity of our work. We have carefully addressed all comments and made the corresponding revisions to the manuscript.

We hope the revised manuscript addresses all concerns and that you find the paper suitable for publication. Detailed point-by-point responses follow below.

If you have any further queries, please do not hesitate to contact us.

Sincerely,

Sandra Montes-Olivas, on behalf of the authors.

Comments from Reviewer 1:

This manuscript investigates the application of direct optimal control methods, particularly nonlinear programming (NLP) using JuMP and IPOPT, to a series of epidemiological ODE models. The authors compare indirect methods (Pontryagin's Maximum Principle, forward-backward sweep) with direct discretization and solve several case studies, including lockdown optimization, "flattening the curve," vaccination strategies, and a dengue transmission model. The paper aims to demonstrate that direct NLP methods are practical, accessible, and flexible for epidemic policy optimization, especially relative to traditional indirect approaches. The topic is potentially valuable, particularly given the increased interest in optimization for epidemic response during the COVID-19 pandemic. The manuscript is well written, and the examples are clear and reproducible. However, the current version reads more like tutorial or software demonstration rather than a research article. The examples rely on low-dimensional "toy" systems, and important issues such as discretization robustness, scalability, and real-world policy constraints need further discussion. The use of extremely simplified models, the absence of rigorous numerical analysis, and the lack of engagement with the real complexities of epidemic control significantly limit the impact. In its present form, the novelty is modest and several essential issues remain insufficiently addressed. Overall recommendation: Major revisions to clarify the manuscript's contribution and acknowledge limitations.

Major Comments

> 1. Clarify the novelty and contribution. JuMP, IPOPT, and direct NLP methods are not new to optimization or epidemiology. The paper's contribution lies in accessibility, reproducibility, and translation to practice. This should be stated explicitly in both the Introduction and Discussion to avoid overstating novelty.

We appreciate the opportunity to clarify the scope and contribution of this work. In response, we revised the Introduction (lines 120–128) to make clear that while direct NLP methods and tools such as JuMP and IPOPT are well established within the optimisation community, their adoption in epidemiological modelling has been limited. We now explicitly state that the contribution of this study lies in providing accessible, reproducible examples that demonstrate how existing compartmental models can be adapted for direct optimisation, illustrate the ease of implementation with the JuMP optimisation framework, and showcase the practical advantages of direct methods for real-time decision-making in epidemiology. We also added an explicit contribution statement to the Discussion section (lines 381–389), emphasising that this work does not introduce new optimisation algorithms but rather demonstrates how established direct NLP methods can be made accessible to epidemiologists through optimisation frameworks such as JuMP.

2. Discuss scalability beyond toy models. All demonstrated models are low-dimensional (SIR variants, a 7-compartment dengue model), deterministic, and idealized. These settings do not represent the challenges that make optimal control in epidemiology difficult (e.g., parameter uncertainty, non-convex feasible sets, delays, spatial structure, stochasticity). The authors must explain why these simplified models are sufficient to support their claims, or else add at least one more realistic high-dimensional example.

We appreciate that the reviewer raised this point and have added a discussion addressing issues of model complexity and scalability (see lines 493–513). We note, however, that the models presented in this study are intentionally based on already established, low-dimensional compartmental frameworks that are widely used in epidemiological research and practice. Variants of the SIR model form the foundation of much of the infectious disease modelling literature and continue to play a central role in policy analysis and decision support [6, 8, 10]. Relatively simple models remain key decision making tools. In addition to very high dimensional simulations, non-spatial deterministic models with relatively few age structures were used for forecasting of COVID-19 cases, with complexity gradually built up over time [4]. The selection of these models serves several purposes. First, their familiarity makes them immediately recognisable to the target audience, facilitating interpretation and lowering barriers to adoption. Second, their widespread use allows for direct comparison with existing results obtained using indirect optimal control methods or simulation based approaches. Third, they demonstrate that the proposed direct NLP methodology can be applied to models already in common use, without requiring fundamental changes to established compartmental structures. While more complex formulations incorporating spatial structure, stochasticity, or population heterogeneity introduce additional numerical challenges, the optimisation framework employed here remains applicable in principle. As discussed in the revised Discussion section, such extensions primarily raise computational considerations, such as increased numbers of decision variables and constraints, potential stiffness, or the need for solver tuning, rather than methodological limitations of the direct optimisation approach itself.

3. Address realism of interventions. Some of the computed optimal controls (e.g., continuously varying lockdown intensity) are unrealistic in practice. The manuscript should acknowledge common policy constraints such as:

1. minimum/maximum intervention durations,
2. delays between decision and implementation,
3. capacity limits for vaccination.

A brief discussion would help contextualize results for policy audiences.

We agree that discussing practical constraints is essential for contextualising the optimal control results for policy audiences. We note that the manuscript already acknowledged some of these challenges in the Discussion section (lines 440–452). However, we have now expanded this discussion (lines 393–400, 463–470 and 595–602) addressing the translation to policy applications in real-world settings.

> 4. Lack of numerical analysis or solver robustness evaluation. IPOPT's performance is highly sensitive to scaling, sparsity patterns, stiffness, constraint curvature, and discretization choices. The manuscript provides no analysis of:
>
> 1. sensitivity to timestep dt,
> 2. solver tolerance settings,
> 3. convergence failures,
> 4. constraint feasibility.
>
> Without this, the claim that the method is "robust" is unsubstantiated.

We thank the reviewer for highlighting this. We have removed unsubstantiated "robust" claims throughout the manuscript and added the solver settings in Methods (lines 170–177), including convergence and feasibility tolerances. We note that all case studies converged successfully with these default settings, with constraint violations remaining below the specified feasibility tolerance, and no convergence failures or manual scaling were required. Sensitivity to timestep is addressed in our response to Comment 5, where we present a timestep sensitivity analysis (see also lines 417–425). Additionally, we acknowledged in the Discussion that, while all presented case studies converged successfully, we have not systematically evaluated sensitivity to initialisation, problem scaling, stiffness, different tolerances, or parameter uncertainty, noting that more challenging problems may require additional numerical considerations (lines 525–548).

> 5. Discretization choice (Euler) is inadequate without justification. Euler's method performs poorly under moderate stiffness or when dt is not extremely small. The authors must justify its use or compare with more appropriate schemes (e.g., RK4, backward Euler, collocation). At minimum, they should report timestep sensitivity.

To address these concerns, we performed a timestep sensitivity analysis for Case Scenario 1 (lockdown model), comparing two discretisation methods: exponential discretisation (using exponential formulation for infection and recovery processes) and simple Euler (linear approximation), across six timestep values ranging from dt =

2.0 to 0.05 days. For each timestep, we examined optimal objective values, convergence behaviour, computational performance (averaging 50 repetitions per optimisation to ensure robust timing measurements), control trajectories, and solution quality. The analysis showed that: (1) both methods converge successfully across all timesteps tested, with all optimisations achieving LOCALLY_SOLVED status, (2) both methods converge to nearly identical optimal solutions at small timesteps (0.599 vs 0.595 at dt=0.05, representing only 0.7% difference), (3) the simple Euler method exhibits better stability across timestep choices, with only 1.58% relative variation from dt=2.0 to dt=0.05, while exponential discretisation shows higher sensitivity (22% relative variation), and (4) computational cost scales approximately linearly with the number of time points for both methods. These results demonstrate that for this non-stiff epidemiological model, the simple Euler method with appropriately chosen timesteps provides reliable results. This analysis can be found in the paper's accompanying repository `https://github.com/epirecipes/EpiPolicies` and visualised in Figure S3, which shows objective value convergence over time, the relative difference between methods, computational time scaling with problem size, and a comparison of control trajectories at dt=0.5.

Previously, we stated that Euler's method was employed as a straightforward and widely used discretisation approach, while acknowledging that it has limitations regarding accuracy and stability. We have now expanded this explanation to include the results obtained (lines 417–425). Additionally, we noted in the Discussion that more challenging problems (e.g., stiff systems, high-dimensional models) may require higher-order discretisation schemes such as Runge-Kutta methods, backward Euler, or collocation methods, and emphasise that the direct NLP framework is fully compatible with any discretisation scheme the modeller chooses to implement (see lines 535–548).

> 6. No comparison with modern control approaches. The Discussion briefly mentions differentiable ABMs but does not meaningfully engage with:
>
> 1. Model Predictive Control (MPC),
> 2. Reinforcement Learning,
> 3. Hybrid direct-indirect methods,
> 4. Deep learning-based optimization (e.g., DQN, policy gradients).
>
> Given current trends, this omission is noticeable.

We appreciate this suggestion. While the manuscript briefly mentions DQN and reinforcement learning in the Introduction (lines 98–102), we agree that it would benefit from a more extensive discussion. We have added a section in the Discussion (lines 549–574) that directly addresses these other approaches. We suggest that direct NLP provides a middle ground between classical analytical methods and data-driven approaches, and that it is complementary to MPC and RL methods, with clear trade-offs

discussed for each approach.

Minor Comments

> Clarify whether parameters in SIR cases were chosen arbitrarily or based on literature.

Thank you for the opportunity to clarify this point. For Case Scenarios 1–3, the manuscript states that the parameter values (listed in Table 1) were assumed for the purpose of intervention optimisation only and were not derived from real-world data (see lines 248–251), while the parameters for Case Scenario 4 were obtained from Asamoah et al [3] (lines 313–314). We hope this clarification addresses the reviewer's concern.

> Add brief comments on solver settings (tolerances, scaling), since IPOPT performance can depend on these.

We have now included the solver settings (tolerances and scaling) in lines 172–174.

> Expand related work beyond a few COVID-era studies; optimal control in epidemiology has a longer history.

We have expanded the Introduction (lines 41–58) to include optimal control applications beyond COVID-19.

> Provide a short statement on how non-convergence was handled (e.g., feasibility issues, solver failure modes).

We have noted that all presented case studies converged successfully, and that no manual scaling or problem reformulation was required (lines 175–177).

Comments from Reviewer 2:

I thank the authors for submitting their manuscript "Exploring epidemic control policies using nonlinear programming and mathematical models". I agree with several of the authors' points in the manuscript, in particular the need for increased use of optimal control theory in mathematical epidemiology and for more approachable methods. The manuscript provides a solid overview of many of the difficulties associated with indirect methods, especially Pontryagin's Maximum Principle, and discussion of how direct methods such as nonlinear programming can provide a useful alternative. However, my enthusiasm for the manuscript is greatly diminished due to its focus on one particular software package and its use of only rudimentary examples of epidemic models.

Major Comments

> 1. In the introduction, the authors highlight only a handful of publications on optimal control of COVID-19. The authors should also discuss work done in other systems, such as vector-borne disease, rabies, foot and mouth. The inclusion of the work done on vector-borne disease control is especially pertinent given that Case Scenario 4 is a model of mosquito-borne disease.

We thank the reviewer for this suggestion. We have expanded the Introduction (lines 41–58) to include optimal control applications beyond COVID-19 with citations for vector-borne diseases [9, 7, 2, 1], rabies [5], and foot-and-mouth disease [11].

> 2. The authors focus almost exclusively on the use of JuMP. While this package has not been used before in epidemiology, it is well established and has been cited several hundred times. Additionally, this software is implemented within Julia, a programming language that is not prominent in the field and would present a significant learning curve to many, if not most, researchers. If the aim of this manuscript is to convince mathematical epidemiologist to move towards direct optimization methods, it would be better to provide an overview and comparison of available packages and approaches across commonly used languages, especially R and Python, beyond simply the computational time.

We thank the reviewer for this observation. We acknowledge that JuMP is used as the primary implementation tool across all case scenarios, with the cross-platform comparison conducted only for the lockdown model. We would like to clarify the rationale for this design choice.

In the revised manuscript, we strengthened the cross-platform comparison (Table 4) by using a smaller timestep ($dt = 0.5$), increasing the number of runs to 100 per

interface, and reporting mean and standard deviation for both overall solve time and function evaluation time. We also included the resulting objective value, confirming that all six implementations across four languages (Julia, Python, R, AMPL) converge to the same optimal solution with the same number of iterations and function evaluations. Since the underlying solver (IPOPT) performs the same numerical computation regardless of the front-end language, the choice of programming interface affects only model specification and automatic differentiation, not the optimisation outcome. We believe that demonstrating this in a single case scenario is sufficient to establish that the methodology can be applied to different programming languages.

JuMP was selected as the primary tool across scenarios because it is fully open-source and offers a concise algebraic modelling syntax that facilitates readability and implementation (see lines 154–164). However, as shown in Table 4, users working in Python can use Pyomo or amplpy, while those in R can use rAMPL, obtaining equivalent results. All implementations are available in the accompanying repository to facilitate adoption in the reader's preferred language. We have also revised the manuscript to clarify that this study aims to demonstrate the practical value and accessibility of direct optimal control methods in epidemiological modelling, rather than to advocate for a specific software package (see lines 119–132).

3. The authors spend much of the introduction comparing direct and indirect methods, but do not compare them within the examples provided beyond showing the derivation of the adjoint equations and Hamiltonian for the simplest model. a. It is important to show that both methods will arrive at roughly the same solution and that the use of direct methods is significantly computationally faster. None of the case scenarios provided (other than possibly case scenario 4) are complex enough to prohibit the use of indirect methods, and comparisons should be provided. b. For readers not familiar with optimal control, it is also important to communicate the difficulty of deriving the adjoints and Hamiltonian with higher dimension models. This could be done by either providing derivations for other scenarios or a discussion of how complexity scales. c. While Case Scenario 1 does compare the optimal solution found by JuMP to another intervention, this intervention is not optimal. This should be made clearer.

We thank the reviewer for their comments. Our intention in including the derivation of the adjoint equations and Hamiltonian for the simplest model was to create an accessible entry point for readers who may be familiar with either direct or indirect optimal control methods, but not both. By presenting this derivation in a simplified context, we aimed to clarify the conceptual connection between the two approaches without overwhelming the reader.

Regarding the comparison between direct and indirect methods, we agree that both approaches can yield comparable optimal solutions for sufficiently simple problems. However, we believe the primary distinction lies not in the quality of the solutions, but

rather in the complexity of implementation and the computational workflow. Even for the simplest cases, the indirect approach requires a non-trivial analytical derivation of the adjoint equations and the development of custom numerical schemes (e.g., forward–backward sweep methods), which results in significant overhead in terms of development time and code maintenance.

In contrast, the direct approach we adopted avoids this overhead by formulating the problem within a unified optimisation framework and utilising readily available, well-tested solvers through JuMP. While the case scenarios we considered could be addressed using either approach, we believe they effectively demonstrate the practical advantages of direct methods, particularly in terms of ease of implementation and access to established solvers.

Furthermore, we verified that both approaches yield equivalent optimal solutions not only for the simple exponential infection model (Figure 2), but also for the most complex model considered in this study. Case Scenario 4 (the dengue transmission model) has now been solved using both the direct NLP approach via JuMP and the indirect forward-backward sweep method, obtaining identical optimal control trajectories. As presented in Asamoah et al. [3], the indirect approach required extensive analytical derivation of seven adjoint equations and four optimality conditions, together with a custom numerical implementation of the forward-backward sweep algorithm. In contrast, the direct approach required only the specification of the model's ODEs, constraints, and objective function, with all derivative computations handled automatically by the solver. The code implementing both approaches and the resulting trajectory comparisons are available in the accompanying repository. We have included this on the manuscript in lines 329–345.

In response to this comment, we have added a paragraph (lines 493–513) that clarifies how the complexity of deriving adjoint equations and Hamiltonians increases with model dimension and explains why this motivates the use of direct methods for larger, more complex models. Also, we have clarified in lines 255–256 and in the Fig.3 description that the intervention shown in Figure 3(b) is a heuristic baseline (intervention at peak) rather than an optimal strategy, to highlight the improvement achieved by the optimisation.

> 4. It would be useful to understand how computational time scales with model complexity. Even Case Scenario 4 is a relatively low dimensional problem. The authors should include computational times for some higher dimensional problems, for instance those that include population structure or multiple pathogen strains.

We agree that understanding computational scaling is important. While we acknowledge that the models presented are relatively low-dimensional, a systematic evaluation of how computational time scales with model complexity is beyond the scope of this work. We address this concern in the Discussion (lines 525–548). Specifically, we recognise that we have not systematically evaluated how computational time scales with model dimension.

The models presented range from a 3-compartment SIR model to the 7-compartment dengue model in Case Scenario 4, which, after discretisation, resulted in 5,409 decision variables and 4,215 constraints (added to lines 520-522). The latter was solved in under 30 seconds using default IPOPT settings. Moreover, for Case Scenario 4 we also implemented the indirect forward-backward sweep method, and obtained equivalent optimal trajectories, confirming that the direct approach produces reliable solutions while requiring considerably less analytical and implementation effort (lines 329-345).

In lines 530–544, we discuss that more complex formulations incorporating spatial structure, stochastic effects, population heterogeneity, or multiple strains may introduce additional numerical difficulties, and that addressing these challenges may require additional numerical measures such as custom scaling, higher-order discretisation schemes, or multiple initialisation strategies. We emphasise that the direct optimisation framework remains conceptually applicable to higher-dimensional problems, though such extensions would require additional methodological considerations beyond the scope of this work.

5. Table 4 suggests that JuMP is actually the computationally slowest of the tested optimizers without the addition of AmplNLWriter, which is a wrapper for AMPL. I'm assuming this would also require the AMPL license, negating the benefit of using JuMP for anyone other than Julia users. Given that JuMP takes approximately 26 times as long with even relatively simple models, this is an important point that is not discussed by the authors and would suggest that it would be better to present one of the other packages, such as Pyomo. If there are important factors that would favor JuMP over Pyomo these should be discussed.

We thank the reviewer for this helpful suggestion. Upon further examination, we found that the larger computational time initially observed for the JuMP implementation was partly attributable to just-in-time (JIT) compilation overhead, rather than solely to the performance of the Ipopt–JuMP solver combination. Accounting for this effect yields a more representative assessment of JuMP's performance (see Table 4 and lines 352–362). We corrected Table 4 to show solve times after JIT compilation warmup, demonstrating that JuMP's performance is comparable to other packages. We also clarified that AmplNLWriter does not require an AMPL license when used with open-source solvers like IPOPT (lines 368–370).

In response to the reviewer's comment, we have also expanded the analysis by including a benchmark study of JuMP with additional solvers (MadNLP and UnoSolver). The benchmark results show that all solvers converged to equivalent objective value for case scenario 1 and case scenario 4, confirming that the solutions are not solver-dependent (see lines 375–379). These results, along with trajectory comparisons, are available in the accompanying repository.

Minor Comments

Variables such as S, I, and C should be referred to as "state variables", not "key variables"

We have corrected this in line 233

The parameters in these models are more properly referred to as transition rates. Probabilities imply stochasticity.

We have removed the term probability of transition.

Lines 317-320: This is a strong statement to make without providing any citations. At minimum, examples should be provided. Otherwise, this statement should be removed.

The wording has been adjusted, and we have included examples to support the statement (see lines 393-400)

Lines 42-42: It is unclear as to what these ongoing challenges are.

We removed "ongoing" to highlight that we were referring to the uncertainty surrounding costs.

Paragraph beginning on line 66: It would be useful to have a more detailed description of what constitutes a nonlinear optimization problem.

We have added a more detailed description in lines 85–92.

References

[1] L. Almeida, J. Bellver-Arnau, Y. Privat, and C. Rebelo, *Vector-borne disease outbreak control via instant releases*, Journal of Mathematical Biology, 89 (2024), p. 63.

[2] J. K. K. Asamoah, E. Okyere, A. Abidemi, S. E. Moore, G.-Q. Sun, Z. Jin, E. Acheampong, and J. F. Gordon, *Optimal control and comprehensive cost-effectiveness analysis for covid-19*, Results in Physics, 33 (2022), p. 105177.

[3] J. K. K. Asamoah, E. Yankson, E. Okyere, G.-Q. Sun, Z. Jin, R. Jan, and Fatmawati, *Optimal control and cost-effectiveness analysis for dengue fever model with asymptomatic and partial immune individuals*, Results in Physics, 31 (2021), p. 104919.

[4] P. Birrell, J. Blake, E. van Leeuwen, N. Gent, and D. De Angelis, *Real-time nowcasting and forecasting of COVID-19 dynamics in England: the first wave*, Philosophical Transactions of the Royal Society B: Biological Sciences, 376 (2021), p. 20200279. _eprint: https://royalsocietypublishing.org/rstb/article-pdf/doi/10.1098/rstb.2020.0279/167863/rstb.2020.0279.pdf.

[5] M. Charles, S. G. Mfinanga, G. Lyakurwa, D. F. Torres, and V. G. Masanja, *A mathematical and optimal control model for rabies transmission dynamics among humans and dogs with environmental effects*, Journal of Applied Mathematics, 2025 (2025), p. 5559842.

[6] H. W. Hethcote, *The mathematics of infectious diseases*, SIAM Review, 42 (2000), pp. 599–653.

[7] S. F. Jaleta, G. F. Duressa, and C. T. Deressa, *A mathematical modeling and optimal control analysis of the effect of treatment-seeking behaviors on the spread of malaria*, Frontiers in Applied Mathematics and Statistics, 11 (2025), p. 1552384.

[8] M. Jit, K. Ainslie, C. Althaus, C. Caetano, V. Colizza, D. Paolotti, P. Beutels, L. Willem, J. Edmunds, B. Nunes, S. Namorado, C. Faes, N. Low, J. Wallinga, and N. Hens, *Reflections on epidemiological modeling to inform policy during the covid-19 pandemic in western europe, 2020–23*, Health Affairs, 42 (2023), pp. 1630–1636. PMID: 38048502.

[9] L. Ouattara, D. Ouedraogo, O. Diop, and A. Guiro, *Analysis and optimal control of a mathematical model of malaria*, Nonlinear Dyn Syst Theory., (2024), p. 24.

[10] M. Shams Eddin, H. El Hajj, R. Zayyat, and G. Lee, *Systematic comparison of different compartmental models for predicting covid-19 progression*, Epidemiologia, 6 (2025), p. 33.

[11] A. Wang, D. Bai, J. He, and S. R. Smith, *Optimal control of bi-seasonal hand, foot and mouth disease in mainland china suggests transmission from children and isolating older infected individuals are critical*, Journal of Mathematical Biology, 89 (2024), p. 43.
