# Supplementary material for: Exploring epidemic control policies using nonlinear programming and mathematical models
Source: PLoS Comput Biol. 2026 May 6;22(5):e1014238. doi: 10.1371/journal.pcbi.1014238 (PMC13170968; doi:10.1371/journal.pcbi.1014238)
Supplement: Attachment — Submitted filename: Montes-Olivas et al - PLOS CB review.docx [file pcbi.1014238.s005.docx]

I thank the authors for submitting their manuscript “Exploring epidemic control policies using nonlinear programming and mathematical models”. I agree with several of the authors’ points in the manuscript, in particular the need for increased use of optimal control theory in mathematical epidemiology and for more approachable methods. The manuscript provides a solid overview of many of the difficulties associated with indirect methods, especially Pontryagin’s Maximum Principle, and discussion of how direct methods such as nonlinear programming can provide a useful alternative. However, my enthusiasm for the manuscript is greatly diminished due to its focus on one particular software package and its use of only rudimentary examples of epidemic models.

Major Comments:

1. In the introduction, the authors highlight only a handful of publications on optimal control of COVID-19. The authors should also discuss work done in other systems, such as vector-borne disease, rabies, foot and mouth. The inclusion of the work done on vector-borne disease control is especially pertinent given that Case Scenario 4 is a model of mosquito-borne disease.
2. The authors focus almost exclusively on the use of JuMP. While this package has not been used before in epidemiology, it is well established and has been cited several hundred times. Additionally, this software is implemented within Julia, a programming language that is not prominent in the field and would present a significant learning curve to many, if not most, researchers. If the aim of this manuscript is to convince mathematical epidemiologist to move towards direct optimization methods, it would be better to provide an overview and comparison of available packages and approaches across commonly used languages, especially R and Python, beyond simply the computational time.
3. The authors spend much of the introduction comparing direct and indirect methods, but do not compare them within the examples provided beyond showing the derivation of the adjoint equations and Hamiltonian for the simplest model.
   a. It is important to show that both methods will arrive at roughly the same solution and that the use of direct methods is significantly computationally faster. None of the case scenarios provided (other than possibly case scenario 4) are complex enough to prohibit the use of indirect methods, and comparisons should be provided.
   b. For readers not familiar with optimal control, it is also important to communicate the difficulty of deriving the adjoints and Hamiltonian with higher dimension models. This could be done by either providing derivations for other scenarios or a discussion of how complexity scales.
   c. While Case Scenario 1 does compare the optimal solution found by JuMP to another intervention, this intervention is not optimal. This should be made clearer.
4. It would be useful to understand how computational time scales with model complexity. Even Case Scenario 4 is a relatively low dimensional problem. The authors should include computational times for some higher dimensional problems, for instance those that include population structure or multiple pathogen strains.
5. Table 4 suggests that JuMP is actually the computationally slowest of the tested optimizers without the addition of AmplNLWriter, which is a wrapper for AMPL. I’m assuming this would also require the AMPL license, negating the benefit of using JuMP for anyone other than Julia users. Given that JuMP takes approximately 26 times as long with even relatively simple models, this is an important point that is not discussed by the authors and would suggest that it would be better to present one of the other packages, such as Pyomo. If there are important factors that would favor JuMP over Pyomo these should be discussed.

Other Comments:

- Variables such as S, I, and C should be referred to as “state variables”, not “key variables”
- The parameters in these models are more properly referred to as transition rates. Probabilities imply stochasticity.
- Lines 317-320: This is a strong statement to make without providing any citations. At minimum, examples should be provided. Otherwise, this statement should be removed.
- Lines 42-42: It is unclear as to what these ongoing challenges are.
- Paragraph beginning on line 66: It would be useful to have a more detailed description of what constitutes a nonlinear optimization problem.
